# Iliac-femoral stent-graft infection after hybrid procedure redo: Case report

**DOI:** 10.1016/j.ijscr.2021.106096

**Published:** 2021-06-08

**Authors:** E. Dinoto, F. Ferlito, F. Urso, D. Mirabella, G. Bajardi, F. Pecoraro

**Affiliations:** a Vascular Surgery Unit, AOUP Policlinico ‘P. Giaccone’, Palermo, Italy; b Department of Surgical, Oncological and Oral Sciences, University of Palermo, Italy

**Keywords:** Infection, Endovascular, Stent-graft, Superficial femoral artery, Common iliac artery

## Abstract

**Introduction:**

Stent-graft infection in peripheral arteries is rare and potentially dangerous. The use of hybrid procedures, in complicated patients previously treated, involves an increase of infective risk especially in no collaborative patients.

**Presentation of case:**

We report a case of rare stent-graft infection in a patient treated for a Rutherford IV Multiple Peripheral Arterial Disease (MPAD) involving the right iliac-femoral axis with stenosis on deep femoral artery due to a previously stenting procedure for Superficial Femoral artery (SFA) stenosis. The first simultaneous hybrid intervention consisted of an endovascular iliac stent-graft placement and a surgical common femoral patch angioplasty. After two months the patient was readmitted to our unit for a purulent secretion through a fistulous channel and a suspect infection of stent-graft. Subsequently, the stent-graft was completely removed without possibility to have a surgical revascularization. An amputation major amputation was needed for irreversible ischemia of right leg.

**Discussion:**

The incidence of stent-graft infection after endovascular aortic aneurysm repair had been reported as 0.4–1.0% while Aortoiliac graft infection occurs in 2–6% of patients. Hybrid procedures are secure and need close follow-up for cases of redo and patient with comorbidities.

**Conclusions:**

Graft infection is a rare complication after endovascular treatments. Hybrid procedures outcomes are good with less morbidity but in patient with high risk of infection is important a close follow-up.

## Highlights

• Stent-graft infection in peripheral arteries is rare and potentially dangerous.
• Stent-graft infection after angioplasty Aortoiliac occurs in 2–6% of patients.
• Hybrid procedures are useful, patients high risk for infection need a close followup.

## 1 Introduction

Multilevel peripheral arterial disease (MPAD) in diabetic patients is a significant cause of amputation. Vascular interventions are required to increase blood flow into extremities to enhance cutaneous oxygen pressure promoting wound healing [1]. Endovascular treatment of peripheral artery disease is increasingly common as an alternative to surgical repair [2]. In case of MPAD, extensive revascularizations have been advocated as determinant to reduce the risk of amputation. Female gender and redo surgery are associated with increased risk of prosthetic graft infections leading to a high rate of limb loss and mortality [3]. However, a lack of cooperation by patient can involve the nullification of procedure with impairment of the initial condition. Herein we report a rare complication of infection in iliac-femoral stent-graft.

This work has been written in accordance with the SCARE criteria [4].

## 2 Case report

A 80-year-old female with hypertension, diabetes mellitus, was referred to our hospital for fever, metrorrhagia and rest pain in right leg. Medical history reported uterine cancer and gallbladder cancer treated many years before with hysterectomy and cholecystectomy, respectively. Five years before, a right superficial femoral artery stenting was performed for rest pain. No coronary artery disease. At admission, her physical examination revealed atrial fibrillation, blood pressure of 150/70 mmHg, fever (37.5 °C), respiratory rate of 20 breaths/min and oxygen saturation of 95%, abdomen's soft and non-tender with inflammation in genital region caused by *Candida albicans* infection with Vescicovaginal Fistula complicated by urinary tract infection. Vascular examination showed lack of distal pulses with cyanosis of right foot. Duplex ultrasound (DUS) showed narrowing residual lumen and monophasic wave on both iliac-femoral axes with occlusion of stent in SFA.

**Fig. 1:**
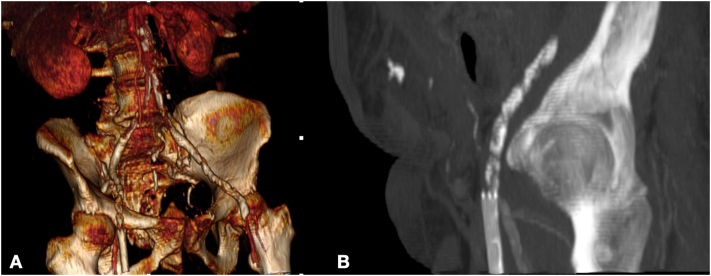
CT angiography 3-dimensional volume rendering (A) and MPR (B) preoperative.

The subsequent CT-angiography showed any significative alteration in the thoracic and abdominal aorta, but in iliac-femoral axes were confirmed an important atherosclerotic disease with significative stenosis on both common and occlusion of right external iliac artery; furthermore, right superficial femoral artery showed an occlusion of stent with its proximal tract involving the origin of right deep femoral artery (DFA), determining an obstacle to direct flow (Fig. 1). After a gynaecological and urological consult that declared as not urgent the problems previously highlighted, the reduced blood flow in the right leg and foot was identified as the main cause of right leg clinical conditions. A simultaneous multilevel hybrid treatment was proposed to address simultaneously the MPAD. After spinal anaesthesia, the hybrid approach consisted of a first step with common femoral endoarterectomy and dacron patch angioplasty prior section of stent involving the origin of DFA. The iliac axis stenosis was also addressed by placing a 7 × 70 mm Silene (InSitu Technologies, USA - Minnesota) in the common iliac artery with extension in external iliac artery with 7 × 150 mm Viabahn Stent graft (WL Gore and Associates, Flagstaff, AZ, USA). After intervention, a direct blood flow was registered in the right DFA. On second post-operative day, the patient was asymptomatic for pain with normalization of cyanosis. The patient was discharged after a week with double antiplatelet therapy and advanced inguinal medication to avoid infectious complications.

After two months of the index procedure, the patient was readmitted due to inguinal wound dehiscence with a purulent secretion through a fistulous channel and signs of sepsis. The patient had missed every check and urological problems were progressed with urine output on the inguinal wound, despite the presence of bladder catheter.

**Fig. 2:**
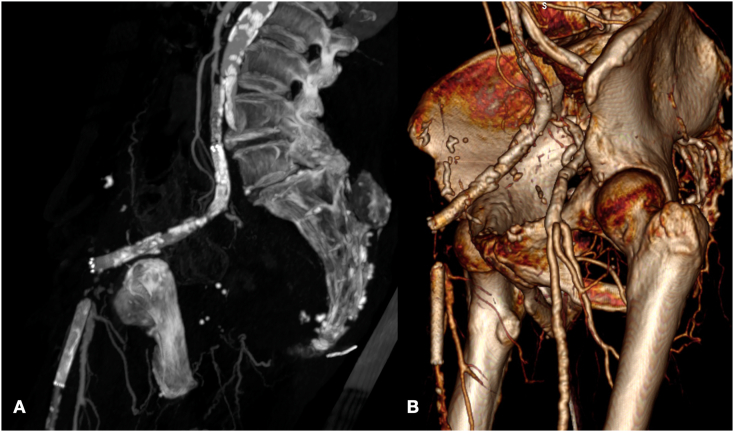
CT angiography MPR (A) and 3-dimensional volume rendering (B) before the second procedure.

**Fig. 3:**
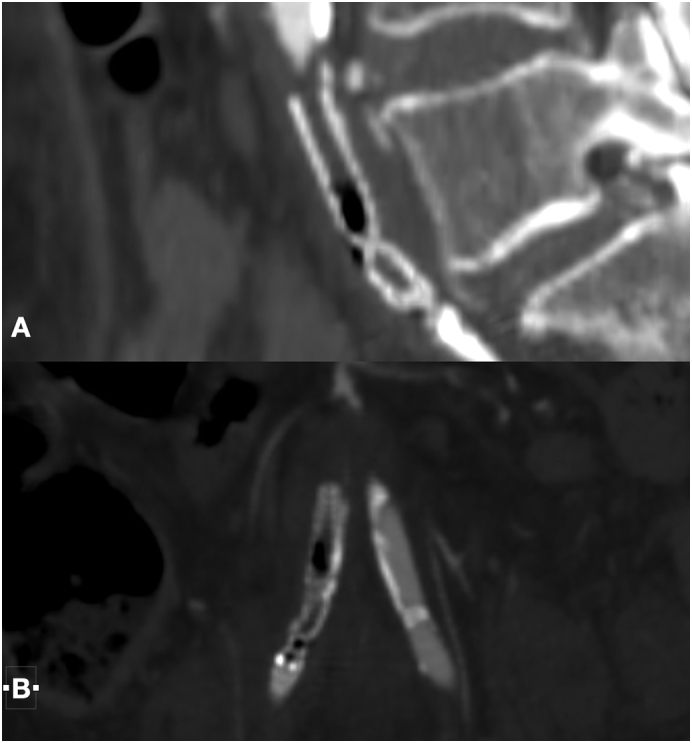
CT angiography MPR with detail of gas bubbles inside the stent-graft in common iliac arteries through the sagittal plane (A) and coronal plane (B).

**Fig. 4:**
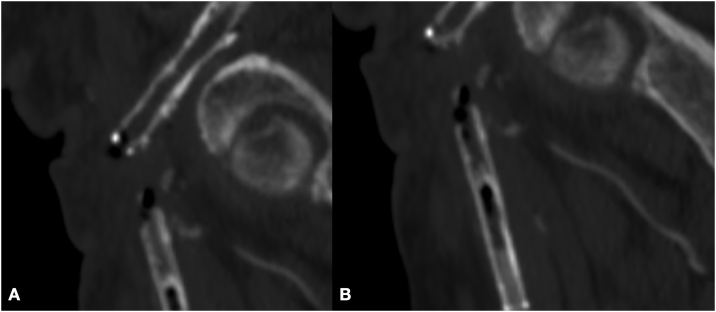
CT Angiography MPR with detail of gas bubbles inside the stent-graft in common femoral artery through the sagittal plane (A) and coronal plane (B).

The control CT showed an occlusion of iliac stent with gas bubbles inside the stent graft and peri-iliac soft tissue attenuation that suggested a graft infection (Fig. 2, Fig. 3). Moreover, there was a suspect of disconnection between patch and arterial wall with dislocation outside of arterial wall in final tract of viabahn stent graft (Fig. 4). Antibiotic treatment was initiated with intravenous meropenem and vancomycin.

Laboratory data were as follows: White blood cell (WBC) 37,000/μL (neutrophils: 95%), C-reactive protein (CPR) 131.5 mg/dl, pro calcitonin 11.6 μg/dl, Hemoglobin (Hb) 8.9 g/dl, Haematocrit (Hct) 27.3%.

**Fig. 5:**
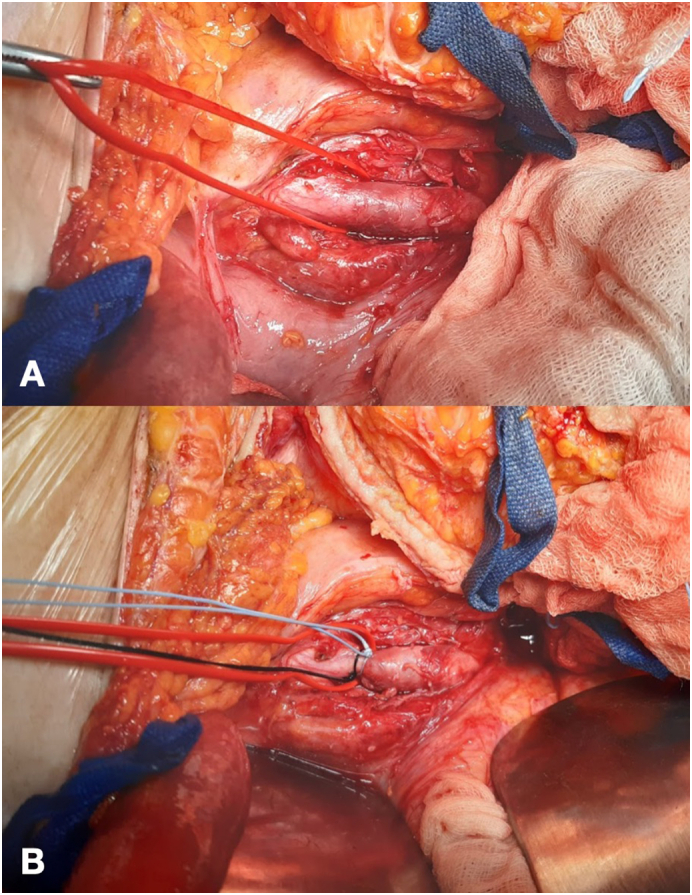
Right common iliac artery was controlled (A) and ligated (B) before arteriotomy.

**Fig. 6:**
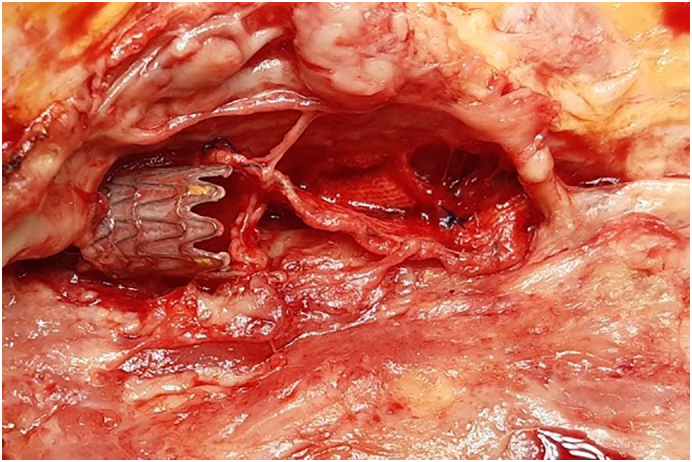
Right common femoral artery with viewing of femoral stent outside of artery with patch disconnected from arterial wall.

**Fig. 7:**
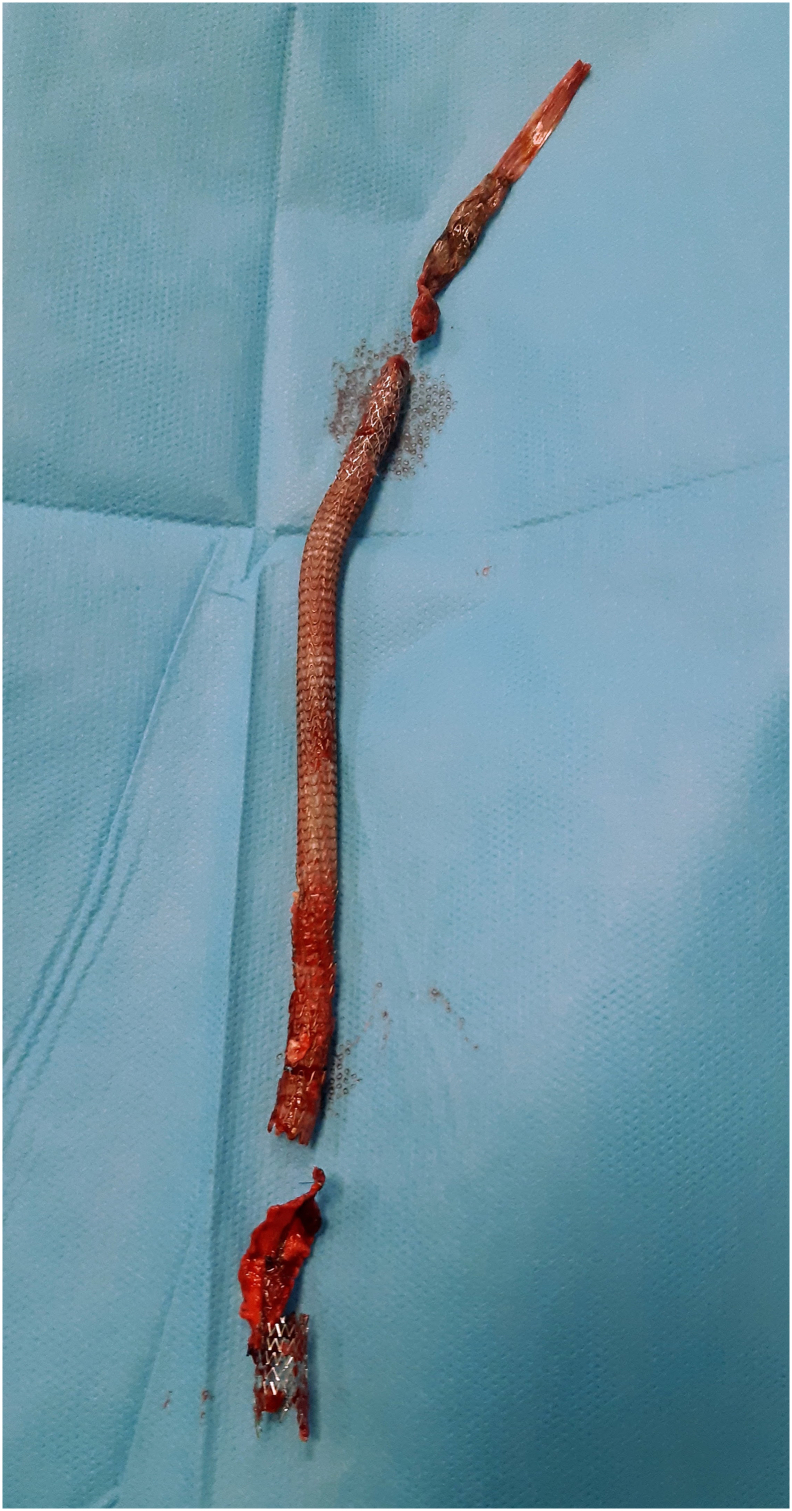
All stent grafts after the removal.

The patient was transferred to the operating room, under general anaesthesia, long midline laparotomy was performed. After peeling off the intraperitoneal adhesion, right common iliac artery was controlled (Fig. 5). In abdomen was present ascites. The stent-graft was completely removed with a traction after a surgical access on common iliac artery and common femoral artery where patch was partially disconnected from arterial wall (Fig. 6, Fig. 7). A procedure of revascalarization was not taken into account for high risk of reinfection and the lack of arterial wall suitable for a vascular anastomosis. Intravenous meropenem and vancomycin were continued for 30 days after surgery, followed by a further 15 days of oral levofloxacin. The cultures from the expulsion site and the explanted stent-graft demonstrated *Staphylococcus aureus* sensitive to the meropenem; thus, the antibiotic regime was continued throughout hospitalization.

At 24 h from index operation, an ischemic deterioration of right leg was present and a major amputation was needed.

After amputation Clinical and laboratory findings were suggestive for sepsis regression: White blood cell (WBC) 20,000/μL (neutrophils: 81%), C-reactive protein (CPR) 108.5 mg/dl, procalcitonin 8 μg/dl, Hemoglobin (Hb) 8.5 g/dl, Haematocrit (Hct) 26%.

The patient was discharged home after 3 weeks with three per day amoxicillin/clavulanic acid 875/125 mg and daily wound dressing. After two weeks, the surgical wound was closed.

## 3 Discussion

MPAD is a common finding in patients presenting CLI. Addressing multiple atherosclerotic lesions is advocated ad determinant to achieve successful clinical improvements [5]. However, endovascular therapies had been increasingly employed especially in high-risk surgical patients with proximal lesions or previously treated with revascolarization procedures [6]. In literature, redo surgery is associated with increased risk of prosthetic graft infections [3]. Stent-graft infection in peripheral arteries is rare but potentially dangerous [7]. The incidence of stent-graft infection after endovascular aortic aneurysm repair had been reported as 0.4–1.0% while Aortoiliac graft infection occurs in 2–6% of patients with such prosthesis [8,9]. Diabetes and smoking independently increase the infection risk; however, other risk factors previously reported for endovascular procedures, such as absence of sterility, lack of antibiotic prophylaxis, introducer sheath permanence more than 24 h, multiple stents implantation, or multiple procedures in the same region [10], and in our case a bare metal stent was present for a precedent FSA stenting. Infected stent and/or stent-graft usually appear as device thrombosis, septic embolization, pseudoaneurysm, and hemorrhage [11,12]. The clinical presentation of our case was an infected fistula with a suspect CT-scan for patch disconnection and stent dislocation outside the arterial wall. Most cases have been described anecdotal in single-case reports. In several publications, the choice of the hybrid procedure combining open femoral endarterectomy with endovascular revascularization in patient to have at first treatment is reported as a safe with no significant differences in infection rates between open surgery, central, or peripheral hybrid revascularization [13,14].

## 4 Conclusions

Graft infection remains a possible rare complication after endovascular treatments. The reduced invasiveness of hybrid procedures determined an increased use in patients presenting multilevel vascular disease and considered at high risk for conventional surgery. Hybrid procedure outcomes are good with less morbidity and shorter intensive care and hospital stay. In patient with high risk of infection or no collaborative is important a close follow-up.

## Ethical approval

None.

## Funding

None.

## Author contribution

Ettore Dinoto: study concept, design, data collection, data analysis, interpretation, writing the paper, final approval of the version to be submitted, guarantor.

Felice Pecoraro: study concept, design, data collection, data analysis, interpretation, writing the paper, final approval of the version to be submitted.

Francesca Ferlito: study concept, design, data collection, data analysis, interpretation, final approval of the version to be submitted.

Francesca Urso: study concept, design, data collection, final approval of the version to be submitted.

Domenico Mirabella: study concept, design, data collection, final approval of the version to be submitted.

Guido Bajardi: study concept, design, data collection, data analysis, interpretation, final approval of the version to be submitted.

## Guarantor

Ettore Dinoto.

## Registration of research studies

Not applicable.

## Consent

Written informed consent was obtained from the patient for publication of this case report and accompanying images.

## Declaration of competing interest

The authors have no ethical conflicts to disclose.
